# Procreating in an Overpopulated World: Role Moralities and a Climate Crisis

**DOI:** 10.1007/s11673-024-10338-y

**Published:** 2024-03-21

**Authors:** Craig Stanbury

**Affiliations:** Monash Bioethics Centre, School of Philosophical, Historical and International Studies, Monash University, Melbourne, VIC Australia

**Keywords:** Procreation, Overpopulation, Climate change, Role morality

## Abstract

It is an open question when procreation is justified. Antinatalists argue that bringing a new individual into the world is morally wrong, whereas pronatalists say that creating new life is morally good. In between these positions lie attempts to provide conditions for when taking an anti or pronatal stance is appropriate. This paper is concerned with developing one of these attempts, which can be called *qualified pronatalism*. Qualified pronatalism typically claims that while procreation can be morally permissible, there are constraints on when it is justified. These constraints often concern whether an individual is motivated to procreate for the right reasons. For instance, if someone is not sufficiently concerned with the child’s future welfare, the qualified pronatalist will say that procreation is not justified. Moreover, David Wasserman says that this concern forms a role-based duty. That is, prospective parents have special duties to be concerned for the child’s future welfare by virtue of the role they occupy. In this paper, I argue that a proper examination of a prospective parent’s role-based duties entails that more is needed to justify procreation. Bringing a new person into the world leaves fewer resources for people who already need them, and the current size of the human population is unsustainable from a planetary point of view. Therefore, even if there is nothing wrong with procreation *per se*, the external condition of overpopulation, and its ensuing public health issues, plausibly gives rise to a role-based duty that prospective parents must account for when deciding whether to procreate.

## Introduction

It is an open question when procreation is morally justified. Antinatalists argue that it is morally wrong to bring a new individual into the world; thus, it is rarely (if ever) permissible to procreate (Benatar 2006; Shiffrin 1999). Pronatalists argue that creating new life is morally good; thus, we should go forth and multiply (Smilansky 1995; Ord 2014). In between these extremes lie attempts to provide conditions for when an anti or pronatal stance is appropriate. This paper is concerned with developing one of these attempts, which can be called *qualified pronatalism*.

Qualified pronatalism typically claims that while procreation can be morally permissible, there are constraints on when it is justified. These constraints are often *internal* because they concern whether an individual is motivated to procreate for the right reasons (Overall 2012; Weinberg 2015). The key question in this context is: Is the person sufficiently concerned with the child's future welfare? If someone answers no to this type of question, the qualified pronatalist will say that the minimal threshold for justification has not been met. David Wasserman (2005, 2015), moreover, says that this constraint means that prospective parents have a *role-based duty* to defend procreation to the child. That is, they have a special duty to the future child by virtue of the role they occupy.

In what follows, I agree with Wasserman that internal constraints form a role-based duty. Contrary to Wasserman, though, I argue that prospective parents’ role morality needs to be further developed to include *external* issues. Bringing a new person into the world leaves fewer resources for people who already need them, and the current size of the human population is unsustainable from a planetary point of view. Therefore, even if there is nothing wrong with procreation *per se*, the external condition of overpopulation may give rise to a moral imperative to limit procreation and have fewer children.

I am not the first to be concerned with overpopulation and the environment (c.f. Rieder 2016; Hedberg 2020; Conly 2016.) Yet, no one has acknowledged that this concern plausibly constitutes a role-based duty. Indeed, remarkably little attention has been paid to developing what a prospective parent’s role morality entails. This lack of attention is problematic because role morality can offer a fruitful way of clarifying and qualifying when procreation is justified. More specifically, it can ground (and reveal) a duty for prospective parents to account for external issues.

Section One explains what role morality is and how prospective parents have distinct roles that yield special responsibilities and duties. Section Two describes the role-based duties and internal constraints that Wasserman places on procreation. Section Three presents the case for limiting reproduction because of environmental concerns. Role morality, I argue, elucidates a duty to defend procreation not just to the child but* to the public.*

## Role Morality

While most moral duties apply to everyone (murder and bullying are generally wrong regardless of who you are), some apply only to people who occupy a particular role (Wasserstrom 1984). Role morality refers to the special responsibilities and duties someone has simply because of their role. Chappell (2019, 194) explains:

A role is a moral, legal, political, institutional or social persona or function or office or guise or *qua* … that brings with it distinctive responsibilities, privileges, powers, immunities, and expectations of the relevant kind.

Once someone identifies as occupying a role, it means they (a) occupy the role, (b) recognize they occupy the role, and (c) acknowledge that the particular responsibilities and duties of the role apply to them (Hardimon 1994). They have (implicitly or explicitly) promised to consider and act on a range of reasons restricted to upholding the role morality of their position (Raz 1986). These reasons form parameters around their conduct. They clarify what the proper norms of the role should be and how failing to meet those norms means failing to meet the moral responsibilities of the role.

Roles can come in a variety of ways. For instance, doctors hold a *professional role*. Occupying the role of a doctor means that an individual is morally required to act in the best interests of their patient’s health (Stanbury, et al. 2024). A doctor has vitiated their professional role morality if they act contrary to this duty. Parents hold a *social role*. No professional code of conduct outlines a parent’s role responsibilities. However, their social position means that they must be partial to and care for their children in a way that others are not expected to.

The source of duties on one’s actions in role morality does not necessarily come from some general account of well-being but from the particular rights and entitlements that can be held against the role. A patient has certain rights against a doctor that constrains what the doctor can do morally; a child has a right against their parents to care for them. Doctors and parents are constrained by what their patients and children are owed. The moral boundaries around what is acceptable within their role are formed by the relationships.

The moral weight of a role-based system of morality could come from its utility value. Doctors fulfilling their role-based duties will likely achieve better patient outcomes; parents caring for their children will probably increase their well-being. However, role morality establishes and reflects important values about a role regardless of the consequences (Wasserman 2008). It shows how people acting in a role should *relate* to others. We value doctors relating to their patients in specific ways—they should be trusting and respect patient confidentiality. A role-based system of morality establishes this value. It shows that anyone who occupies this role has the prerogative to seek trust and be worthy of it (Rhodes 2020). Likewise, we value parents being partial to and caring for their children. Attaching this duty to the parental role elucidates and grounds this value.

In this paper, I am concerned with developing the role morality of a *prospective parent.* Being a prospective parent may appear to be an odd type of role. It may seem that whatever special duties apply to a parent also apply to a prospective parent. However, there is a logical and conceptual distinction between the two. They are in different moral positions because their relationships with those affected by their actions vary. Parents, for instance, have special responsibilities to feed, clothe, and raise their children in a way that prospective parents cannot have. Prospective parents, as we will see, have duties to *intend* to love and care for their now-unknown child in a way that parents (with *actual* children) cannot do. They must also morally engage with whether they should become a parent in the first place. Parents (who already have a child) cannot do this.

The prospective parent role can be a slippery one to grasp. I follow Wasserman by using it to refer to anyone in the position of considering, attempting, seeking, wanting, or in the process of becoming a parent. It can be unclear at times when a prospective parent becomes a parent, particularly concerning embryo and foetal development. It can also be unclear when someone is occupying a citizen or prospective parent role. Nevertheless, nothing in my (or Wasserman’s) argument hinges on the boundaries between roles being sharp. Instead, the point is that while the distinction between roles can be blurry *at the edges*, there is a distinction.

My conceptualization of the role is consistent with the literature.^1^ Most references to prospective parents denote people deliberating whether, when, or how to have a child. Yet, while it is a recognized moral position, the role-based duties that follow have not been adequately clarified. With the notable exception of Wasserman (2005, 2008, and 2015), developing a prospective parent’s role-based duties has mainly been ignored.

Other principles affirm the existence of responsibilities for people wanting to have children, but many of these are not role-based or are slightly different from a justificatory point of view. For instance, the distinguishing feature of a role morality account of justified procreation is that it is person-affecting: prospective parents are constrained by the rights *of* (and duties *to*) those affected by their decisions. It frames the impermissibility of procreation in a particular way: it is wrong *because of* how prospective parents ought to relate to those they are associated with. The relational dynamic between prospective parents and others establishes and elucidates exactly what they owe and to whom they owe it.^2^

However, principles such as Savulescu and Kahane’s (2009) Procreative Beneficence or Buchanan et al.’s (2001) Principle N are impersonal: acting wrongly is not wrong, for example, *to* the child.^3^ Impersonal standards—such as the overall good or value that the child realizes and contributes to the world—determine the rightness or wrongness of procreation.

External facing principles such as Douglas and Devolder’s (2013) Procreative Altruism or Saunders’ (2017) Principle of Generalized Procreative Non-Maleficence also assume more generalized standards of morality—promote the well-being of others or do no harm—to claim what prospective parents should do. Moreover, each of these above principles are concerned with *same-number* choices: procreative choices that do not affect the population number. From the assumption that a child will be born, they develop stipulations around which embryo a prospective parent should select. The procreative choice that I am most interested in is a *different-number* choice: whether to have a child. Role morality helps to address this concern because it can be applied to either same-number or different-number choices.

So, while noting that role morality is not the only word on procreative decision-making nor the only inroad into conceptualizing justified procreation, understanding prospective parents’ role-based duties is an important moral task. Understanding who has rights and entitlements against the role elicits clarity, and going against these rights requires strong justification. Addressing a prospective parent’s role morality, therefore, can offer meaningful insights into when procreation is justified.

Wasserman provides a helpful start. He argues that defending procreation to the child ought to be regarded as a role-based duty. Anyone acting *qua* a prospective parent is vitiating their role responsibilities if they fail to consider the good of the future child when wanting to procreate. A prospective parent is doing something wrong—they are likely unjustified in procreating—if they do not satisfy this role-based duty. In Section Two, I present and agree with Wasserman’s argument as far as it goes.

Section Three claims that his argument does not go far enough. Properly examining a prospective parent’s role morality reveals a duty to factor in overpopulation and climate change when considering procreation. A procreative act that fails to do so does not comply with what is owed of them.

## Defending Procreation *To* The Child

According to Wasserman, anti-natalist arguments do not show that procreation is necessarily wrong; they only show that it needs a defence. He agrees that procreation is morally risky and requires serious reflection but that the harms of procreation do not always outweigh the benefits. A prospective parent can justify procreation by fulfilling their role-based duty to defend their decision *to that child* to bring them into the world:

The actual child is entitled to a respectful reason for having been brought into a world where she is exposed to the harms and risks so vividly described by the anti-natalists. Her progenitors must have intended to have a child in part so that he or she could enjoy a life whose goods would outweigh those bads. (2015, 200)

For Wasserman, procreation can be justified via two related reasons. One, to give the goods of life to a now-unknown child. Two, to form an intimate relationship with a child to confer those goods. Including these reasons in the decision to procreate is a role-based duty. Failing this duty means that procreation is unjustified. These reasons, he says, allow prospective parents to acknowledge the risks and costs of procreation to a future child. Yet, they *also* enable them to claim—if the child were ever to ask—that the harm of life will be plausibly offset by the goods that the child is expected to enjoy. By seeking the good of a future child, prospective parents can bear children for reasons in favour of those children.^4^

Wasserman says that acting for reasons in favour of a now-unknown child works in a similar way to acting for reasons in favour of a now-unknown partner. While it is fine to have selfish reasons to seek a relationship—such as one’s own happiness and fulfilment—one should be motivated at least in part for reasons that concern the good of the future partner. A prospective partner should only seek a relationship if they intend to care about the other person. To justify entering a new relationship—whether that be with a now-unknown child or a now-unknown partner—*their* well-being ought to be a consideration.

At least two consequences follow from Wasserman’s account. First, if the prospective parent cannot cite reasons that concern the good of the future child for procreating, they are vulnerable to a complaint from the child for any hardships they face. Wasserman (2005, 151) says: “Because the goods of that child’s life were no part of their reason for having him, they cannot adduce them to offset or justify that hardship.” Procreating *solely* for selfish reasons does not satisfy the prospective parent’s role-based duties to the child.

Second, if prospective parents believe the child’s prospects of receiving “the goods of life” are negligible or do not think they can be a good parent, they are not justified in procreating. There may be a threshold risk of oppression or suffering for a new child below which one has a responsibility not to bring that child into the world. The risks to the child may be extreme, the suffering likely, and the hope for improvement not realistic enough, such that procreation is unjustified.

I agree with Wasserman that being motivated to procreate for reasons that concern the good of the child ought to be a role-based duty. If a prospective parent has a duty to defend procreation to that child, it will likely result in better outcomes for the child. It increases their chances of being born into a loving environment where their interests and eventual autonomy are respected. But, regardless of the consequences, it establishes how an occupier of the role should relate to their future offspring. Just as a patient has rights against their doctor, a child should have rights against their future parents to be concerned about their welfare. The suffering the child will one day experience is morally relevant, and attaching a duty to the role to consider this suffering reflects that.

This duty is role-based because it elucidates what rights and entitlements can be held against anyone occupying the role of prospective parent and thus what duties they are constrained by. Moreover, it is *particular* to prospective parents. Concern for the future well-being of their now-unknown child is a responsibility only a prospective parent can have. Someone not deliberating about bringing a person into the world—a *non*-prospective parent—has no responsibilities to this end. A parent (whose child already exists) can also not have this responsibility.

There is likely reasonable disagreement about the precise rights and entitlements the future child can have on a prospective parent (for instance, Battisti 2023 argues that Wasserman sets the standard for justification to the child too low). Entering into this debate is not my focus. I remain neutral on some specifics of a prospective parent’s internal duties to the child and simply agree that the child has rights against the prospective parents, and so there *are* such duties.

My concern—and the critical point I want to make—is that while Wasserman has offered important insights into justified procreation, he has not captured the extent of a prospective parent’s role-based duties. He says nothing about how the role should relate to third parties and establishes no values about its position in society.

This is a problem. I will argue that future children are not the only ones deserving of rights and entitlements from prospective parents. Given that procreation has significant implications for others in the context of climate change and overpopulation, it is reasonable to require prospective parents to consider the impact of their procreative choices on others. Their role morality should be expanded to include concern for the external issues associated with bringing a new person into the world. To my knowledge, no one has yet acknowledged this as a role-based duty.

## Defending Procreation *To* The Public

Wasserman acknowledges that external issues *could* be a factor in deciding whether to procreate, but he thinks that this acknowledgement is separate from a prospective parent’s role morality:

Prospective parents, like actual parents, are entitled to discount and even ignore some consequences of their decisions for third parties. Specifically, neither are required to universalize in making those decisions; to be constrained by the cumulative impact if “everyone” chose as they do. [2015, 251]

He goes on to say that: “prospective parents whose decisions threaten substantial adverse social effects may have an all-things-considered reason to “break role” in deference to their duties as citizens” (2015, 254). For Wasserman, such concerns are attributable to citizens but do not specifically form part of the moral duties of prospective parents *as* prospective parents: “I do not think they must take account of concerns about population size … in their procreative decisions” (2015, 255). Wasserman admits to not offering a complete argument to this point other than claiming that taking into account third-party interests is not critical to the practice of parenting (or procreating) as we (or he) understand(s) it. He says that doing so “would be at odds, both morally and psychologically, with the parental role that succeeded it” (2015, 252).

These comments by Wasserman can be placed in a broader discussion in procreation ethics about whether the decision to have a child is too personal for external issues to be a factor—let alone an *obligating* factor. Robertson (1994, 24), for instance, says that procreation should have “presumptive primacy when conflicts about its exercise arise because control over whether one reproduces or not is central to personal identity, to dignity, and to the meaning of one’s life.” Rieder (2016) argues that prospective parents should move towards a small family ethic because of overpopulation and climate change. However, he stops short of claiming that they are *obligated* to.

On the other hand, Hedberg says that anyone who believes they should not be environmentally reckless has a moral obligation to limit their family size: “If there is an obligation to reduce one’s unnecessary greenhouse gas emissions, then people should also limit the size of their families” (2019, 1). Conly (2016) claims that because of climate change worries, once people’s fundamental interest in procreating is satisfied by having one child, there is a duty not to have more.

Thus, it is an unresolved question in procreation ethics whether and to what extent prospective parents are morally obligated to consider external issues in their procreative choices. Is there a *duty* to be less pro-natal because of these issues? If so, how can this duty be grounded? Role morality, I believe, can contribute to this discussion. Contrary to what Wasserman says, the relational dynamic between prospective parents and society entails that the public very plausibly has rights against prospective parents that constrains when procreation is justified. Specifically, role morality elucidates (and grounds) a duty for prospective parents to consider overpopulation and the environment when deciding whether to procreate. These issues are so serious that they are now critical to the practice of procreating. Wasserman’s notion that public-facing concerns “break role” does not acknowledge this.

Before pressing my case, I will briefly clarify my target and narrow my scope. The following is primarily aimed at prospective parents *in high-emitting countries*. I will work under the assumption that the higher the emissions one’s child will produce—or the more resources they will consume—the more accountable to the public prospective parents should be for their procreative decision. It may be the case that as further countries develop out of poverty, and thus consume an increasing amount of resources, a role-based duty to the public will become applicable to more prospective parents. Either way, as it stands, many women in countries where fertility rates are high but per capita emissions are low lack adequate access to contraception. It would be unfair to apply my argument to those who cannot take control of their fertility. Anytime I refer to prospective parents from here on in, I am primarily referring to those from high-emitting countries.

I also recognize that “the public” is somewhat vague. I use it to refer to anyone in the broader global society who is or will be affected by (i.e. a victim of) climate change, resource issues, and the unsustainability of the human population—from both current and future generations. That said, while almost everyone will be affected by climate change, the first victims and the most affected are sadly (and all too often) those from low-emitting countries. This is unjust. Perhaps, then, particular attention should be given to them when considering the public.

### Overpopulation

To better understand why prospective parents from high-emitting countries deserve particular moral scrutiny, consider the distinction between “overpopulation” and “population growth.” Overpopulation does not imply nor is it synonymous with population growth. Just because a population might be stabilized or even declining does not entail that it is yet a sustainable size. It may be sustainable, or it may still be too large (Kuhlemann 2018). For instance, if everyone lived like people in affluent countries——such as the United States, United Kingdom, Australia, or places in Western Europe—there would need to be over four Earths to sustain the global population (Global Footprint Network 2023). These places, therefore, are overpopulated even if their fertility rates are not growing.

Indeed, Pimentel, et al. (2010) show that if everyone lived like the average Western European, the global population would need to be reduced to about *two to three billion*. They reach this conclusion by examining the limits of the Earth’s natural resources. The cropland required for a person to live like a Western European is around half a hectare. The average cropland available is only about 0.22 hectares per person.

DasGupta (2019) reaches a similar conclusion via an economic lens. He calculates what global average income level would be sustainable. Average income, in this instance, is a proxy for consumption levels: the higher the income, the higher the consumption. If the global average income is $20,000 per year, the Earth could only sustainably support about three billion people. As income levels rise, the number of people the Earth can sustain within its natural limits lowers. Given that over eight billion people are currently on the planet, anyone earning over $20,000 is not living sustainably.

Other calculations by Crist (2019), Tucker (2019), and Lianos and Pseiridis (2016) reach the same conclusion: regardless of affluent countries’ stabilisation or projected population decrease, many of them will remain overpopulated because the global population needs to be *more than halved* to sustain them. By procreating, they are putting the Earth in an ecological overshoot.

For prospective parents living in the affluent West, this has ramifications. Firstly, it entails that having a child *requires* many others to live in abject poverty: creating a child who will produce high emissions is incompatible with many other people doing the same. Secondly, and therefore, procreation *depends* on injustice and inequality: it relies on having opportunities to flourish that others will not have (Rieder 2016). Such a situation is not commensurate with what the goals of humanity should be. Overall (2012, 177) puts it well:

Humanity’s long term goal must be, at the very least, to achieve a population size compatible with our continued existence on this planet. But that compatibility must surely be such that human beings do not merely survive but also thrive, and not just some but all of us.

One way to address this issue might be for the *whole world* to decrease its per capita emissions. However, on its own, this will not be enough. Adequately responding to climate change and sustainability problems requires addressing population size. Consider, for instance, the Paris Climate Agreement. This agreement aims to avoid catastrophic climate change by preventing the world from warming by 2℃ from pre-industrial levels. To reach this goal, global emissions must be halved by 2030, halved again by 2040, and then *again* by 2050 (Rockström, et al. 2017). It is almost impossible to do this without reducing the population.

First, there is not enough evidence to suggest that affluent nations are willing to (or can) reduce their per capita emissions enough to secure a sustainable future (IPCC 2023). To even stabilize global emissions at their current levels while keeping pace with population, per capita emissions must decrease by 1.2 per cent *per year*. As a global population, we have not managed to achieve a decrease of even 1 per cent over the past fifty years *in total* (Meyerson 2008). Second, developing countries must be allowed to increase their emissions to escape poverty. Being in poverty means that minimal resources are consumed. To stay at this low level of consumption is dehumanizing. So, rather than wanting many people to decrease their per capita emissions, we should be advocating for them to consume *more*.

Third, even if the world’s average per capita emissions did decrease, the number of people on the planet multiplies emissions. Population is responsible for about three-quarters of the global increase in emissions, with only one-quarter attributable to a rise in per capita emissions (Gerlagh, et al. 2018). Global emissions also tend to track one-to-one with population size. Between 1975 and 2009, for example, population and emissions rose by 43 per cent in the United States (Ryerson 2010). Not addressing population size, therefore, means that we will undo any of the good work achieved by reducing per capita emissions.

Fourth, if global fertility rates dropped by only a further 0.5 births per woman (to the U.N.’s “low fertility” population scenario), almost a third of the emissions needed to avoid catastrophic climate change could be saved. This slight drop is equivalent to the annual emissions that would be saved from doubling the fuel efficiency of cars, increasing wind energy fifty-fold, or improving nuclear energy three times over. It accounts for over half of the earth’s yearly emissions (O’Neill, et al. 2010). Thus, fewer children—particularly in affluent countries—and a less overpopulated world will reduce the amount of carbon that will enter the atmosphere, ultimately helping not just short-term climate goals but also long-term ones. Indeed, population size, as Kuhlemann (2018, 184) says, always matters:

A smaller population size will mean a smaller environmental impact, slower resource depletion and a greater range of alternatives for coping with resource scarcity… Conversely, a bigger population will have a greater environmental impact, a faster rate of resource depletion, fewer alternatives for coping with scarcity owing to the concatenation of multiple scarcities and to greater competition for resources, and a greater number of human lives at risk than would otherwise be the case. Population size *always* matters, and in today’s world, a smaller population is a more resilient one [her emphasis].

On top of this, we cannot address per capita emissions without addressing reproduction. The lifelong decision to not bring someone into the world is about twenty times more effective at reducing individual emissions than the *sum* total of many other “green” acts, such as recycling and driving less (Murtaugh and Schlax 2009). In a high-emitting country, having one fewer child saves about fifty-eight tonnes of emissions per year. The next best decision one can make to limit their emissions is to live car-free. But, this will only save about 2.4 tonnes of emissions per year. Furthermore, a family in the United States who opt to have one fewer child achieves the same emissions savings as 684 teenagers who comprehensively recycle for the rest of their lives (Wynes and Nicholas 2017). Procreating, therefore, is the single worst act an individual (qua individual) can do to the environment.

The upshot is that failing to address overpopulation and the attendant overshooting and sustainability problems compromises many people’s access to fundamental human rights such as food, water, shelter, and security, both now and well into the future. Not achieving the Paris Climate Agreement’s goals will likely contribute to tens of millions of deaths in the twenty-first century, billions of deaths over the current millennium, and an unquantifiable amount of suffering that does not result in death (IPCC 2023). As Pimentel et al. (1994, 364) state: “to do nothing to control population numbers is to condemn future humans to a lifetime of absolute poverty, suffering, starvation, disease, and associated violent conflicts as individual pressures mount.”

So, insofar as humanity values sustainability and the capacity or opportunity to flourish for *everyone*, the global population needs to be reduced. Reducing the population by culling people who are already alive is untenable. It must be done by having fewer children. It seems reasonable, then, that the public can legitimately have rights against prospective parents, particularly from high-emitting countries, to factor in these issues when considering procreation. It is a morally relevant feature of their decision. When having a child contributes to further injustice, it must be accounted for.

### A Role-Based Duty *to* the Public

The above is not offered as a knockdown or exhaustive argument. My aim is milder: to provide reasons which show that prospective parents very plausibly have a role-based duty to—*at the very least*—consider the public when deciding whether to procreate.^5^ Procreation is morally significant not just because it could cause harm to one’s child but because of environmental, overpopulation, and justice problems. With millions of people in dire poverty, fragile ecosystems, and the adverse impact of overpopulation on the environment, a prospective parent’s properly developed role morality should reflect these issues.

Developing a role-based duty to the public establishes important values about what the role *is* and how an occupier of the role should *relate* to society. Failing to develop this role-based duty fails to adequately contextualize procreation as something that does not just occur in isolation from one’s community and needs. Instead, the decision to bring someone into the world is set against the backdrop of overpopulation and climate change. Bearing children is not just something a prospective parent must be able to afford, but the planet, too. Therefore, prospective parents have, I believe, a responsibility to be concerned with the sustainability of their actions. The number of resources available for everyone to have the opportunity to flourish should be a factor in their decision-making.

The basis for this role-based duty is not one of attempting to maximize the good. Rather, it is based on the rights and entitlements that the public can legitimately hold against a prospective parent. Climate change and overpopulation are such serious problems that it causes us to re-evaluate and re-think what the role means and how we understand it. Just as a patient has rights against a doctor and a child against their parents, so too, I argue, the public ought to have rights against prospective parents. These rights form constraints around their actions that qualify when procreation is justified.

Nevertheless, some may object here. Some may say that while having a child does indeed contribute to overpopulation and cause more emissions relative to anything else I (qua an individual) can do, global warming will happen (or not) independently of my actions. Whether *I* have zero or ten children will not affect population trends enough to impact global warming. Individuals should not be obligated to reduce their emissions because climate change is a global, intergenerational crisis that requires broad and systematic institutional change (Gardiner 2019). It might be commendable that a prospective parent considers overpopulation and climate change—it might be encouraged or praiseworthy—but it should not be a role-based *duty*.

While this objection has compelling aspects, it misses some morally relevant points. First, even though one person cannot *cause* climate change, we have responsibilities not to contribute to it. The cumulative effect of procreating in an overpopulated world contributes to an unsustainable future. Hence, it is not enough to think of only *one’s* impact. Instead, prospective parents must conceive of themselves as part of a collective which causes damage. The cumulative effect of bringing high emitters into the world is unsustainable. People recycle for similar reasons. Individuals do not recycle because they believe *their* recycling will save the planet but because they believe it is good not to contribute to harming the environment (Rieder 2016). At a collective level, if no one recycled, then consumption would be even more unsustainable. Since procreating is worse for the environment than not recycling, a duty to the public appears plausible.

Second, without having the scope to wade too far into the complexities of intergenerational justice, and notwithstanding issues surrounding the non-identity problem, it seems reasonable that prospective parents should be particularly concerned with the climate injustices that future generations will face—the injustices suffered by the people *they* decide to bring into the world.^6^ If climate change causes massive harm, future generations will carry burdens that the present ones do not. This causal effect creates special obligations for prospective parents. Indeed, the chances of defending procreation to the child will decrease as it becomes less likely that the benefits of life for them will outweigh the harms. If this is the case, prospective parents cannot consider the child’s future well-being (i.e. their internal role-based duty) in isolation from public issues (i.e. their external role-based duty). Concern for the former may entail consideration of the latter. Either way, prospective parents are in the unique moral position of contributing to overpopulation *and* creating the people who will experience the problem.

Third, regardless of the consequences, prospective parents have duties of *justice* to the public. As mentioned above, a person born in the developed world can only live their high-emissions lifestyle if there are either (a) no more than two or three billion people on the planet or (b) more than that but other people living in abject poverty. With (b) being true, many prospective parents have duties to the public not to be part of an unequal and unjust system. An affluent family may be confident that *their* child will be in the fortunate two or three billion and never suffer the consequences of overpopulation and climate change. But, justice demands that they consider how bringing someone into the world requires others not to have the same opportunities as their child.

Fourth, individuals procreate, not governments or institutions. Overpopulation is a public health crisis, and prospective parents are uniquely situated as the ones who can alter this issue. Whether more emitters are created—whether the population rises—ultimately depends on individuals’ decisions. This point remains true *without* abdicating governments or broader institutional responsibility to combat climate change. Nothing I have said mitigates or precludes the importance of a government’s role towards the environment. It does not even claim that prospective parents are primarily responsible for fighting climate change. I am merely highlighting that regardless of any moral duties attached to *other* roles, prospective parents have a role-based duty concerning the environment.

With that said, someone may still object that living in an affluent country where the fertility rate is below replacement levels actually gives people a duty to consider *having* children. Climate change and overpopulation are not the only factors that affect well-being. Having a child, for instance, may improve the dependency ratio or generate economic benefits.

However, while other factors no doubt contribute to well-being, there is likely an asymmetry between duties to have children and duties not to have children (c.f Overall 2012). One way of conceptualizing this asymmetry is to consider again the analogy with entering into a relationship. In a liberal society, most people would agree that there is no duty to enter into a relationship with someone regardless of how much it might increase that person’s well-being. Yet, once someone *does* decide to enter into a relationship, certain constraints apply because of their partner’s rights against them. The same, I think, goes for prospective parents and procreation. There is likely no duty to become a prospective parent in the first instance, but once someone decides to put that hat on, so to speak, there are certain rights that constrain their behaviour.

In any case, there is a distinction between having a duty to do something and having reasons to do that thing. Economic issues can, in some circumstances, count as *reasons* for having children. But, I remain neutral on whether there is a *duty* to factor them in when considering procreation. If there is such a duty, I am simply committed to prospective parents still needing to weigh the role-based climate duty against any other duties. Nothing about the role-based duty I argue for precludes factoring in other issues. The point is simply that it must be accounted for.

All things considered, then, I maintain that it would be remiss to sheer off a duty to the public regarding overpopulation and climate change from a prospective parent’s role morality. The public is entitled to a response from prospective parents that shows they have, at the very least, factored these issues into their decision-making.

## Concluding Remarks

Enriching the role morality of a prospective parent is a helpful way of clarifying and grounding what they are *obligated* to do. It frames justified procreation in a particular way. That is, it shows who has rights against a prospective parent and thus what duties they have to others.

I do not claim that role morality is the only way to approach the ethical challenge of how prospective parents should act. But, insofar as role-based duties have an important place in moral reasoning, understanding what they entail offers meaningful insights. There is a moral difference between procreation that falls *within* the role’s duties and procreation that falls *outside*. If it falls outside, then it takes strong justification to show why the rights that can be legitimately held against the role, and so the duties to others that they are constrained by, should be vitiated.

Claiming that the public has rights against prospective parents is no mere quibble. When deliberating about whether or when to have a child, most people only consider the internal constraints: Can I be a good parent? Will the good of life offset the harm? If they answer “yes” to these questions, they usually take their role-based duties to be fulfilled. Moreover, upon hearing that someone else is pregnant, we often consider it justified if they have answered “yes” to the above questions. We do not typically ask them whether they considered the environmental consequences of procreating. But role morality shows that we should. Anyone acting *qua* a prospective parent is not just encouraged or recommended to care about overpopulation and climate change; they have a role-based duty to do so—their role grounds an *obligation* to.

In sum, what I have argued is by no means the final word on the topic. No doubt much more needs to be said. For instance, the task of fleshing out how *exactly* to factor in overpopulation and climate, and so what questions prospective parents need to ask themselves, still needs to be undertaken. I have merely attempted to sketch the outline of a position and only hope that this sketch invites further discussion. On my account, a fruitful way of determining justified from unjustified procreation is whether it is compatible with a prospective parent’s role morality. And, role morality very plausibly reveals that prospective parents must consider overpopulation and climate change when deliberating whether to procreate. The public is entitled to this consideration.

## Footnotes

1 For example, see Wasserman (2005, 2008, and 2015), Vehmas (2001), McDougall (2005), Weinberg (2015), Trisel (2012), DeGrazia (2012), Wilkinson (2010), Hedberg (2019), Rieder (2016), and Rulli (2014) who all conceptualise prospective parents in a similar way. At times throughout the literature, “prospective parent” is used synonymously with “would-be parent,” or “potential parent.” I do not have a preference in terminology, so use “prospective parent” to stay consistent with the most common usage.

2 Insofar as an account of justified procreation reflects this, it could be considered a role-based duty. For example, Buller and Bauer’s (2011) minimal threshold account which claims that, when selection is possible, prospective parents are required to select the child who is expected to have a life worth living over one that does not (any embryo that satisfies this criterion is acceptable), could perhaps be role-based if the reasons for not selecting a child below the threshold are person-affecting. That is: it is concerned with how it wrongs *the child*. Battisti’s (2021) Bold Restriction of Procreative Autonomy and the Mild Restriction of Procreative Autonomy is person-affecting and says that even if a child is expected to have a life worth living, if they have a disease that could significantly impair their psychological or physical well-being, then they have a right to complain to their parents. Nevertheless, these two principles (as framed by the authors in their arguments), and the ones that I go onto mention in the next paragraph of the main text, only concern *same-number* choices. I say more about what this means in the main text but essentially, being either same-number or different-number is not a necessary feature of role morality. It can be applied to both. And, this paper is concerned with different-number choices.

3 For instance, if A and B are numerically distinct individuals and so will have different lives, and both lives are expected to be good, yet, A’s life is expected to be better than B’s, selecting B over A—going *against* the demands of procreative beneficence—cannot be wrong *to* B, but only wrong according to some account of overall welfare.

4 Wasserman uses the role morality of prospective parents to argue *against* the principles according to which prospective parents have a duty to select the “best off” child (such as procreative beneficence) or select against a particular characteristic (such as Principle N). He says that all a prospective parent’s role morality requires is that “if people bring children into the world, they do so in part for certain reasons, reasons that concern the good of those children. All prospective parents should expect their children to face significant hardships—death, loss, frustration, and pain—that dwarf the specific hardships associated with most impairments. They must be able to justify the decision to subject their children to those hardships, and they can do so only if part of their reason for having those children is to give them lives good and rich enough to offset or outweigh those hardships” (2005, 135). Therefore, according to Wasserman, a prospective parent does not have any duty to select the “best off” child or select against a particular characteristic as long as they satisfy the above role morality. However, while he uses role morality to make claims about selective reproduction, I remain neutral on the matter. Indeed, for the sake of moving my argument forward to its main claims, I merely commit to agreeing with the minimum claim needed to satisfy one’s role morality, not any potentially attendant consequences regarding selection.

5 While I have deliberately limited my claim to saying that prospective parents have a role-based duty to *consider* overpopulation and the environment, I am open to expanding this duty. I applied this limitation simply because my main goal is to show that, contrary to Wasserman, the external issues should be brought into the fold of a prospective parents’ role morality. The task of spelling out the details further is beyond our scope.

6 Regarding the non-identity problem, for some it is debatable that we can have duties toward future generations given that their identity depends on our actions. I do not have the scope to properly address this problem and so will simply assume, following Cripps (2022), that basic justice demands that we care about the well-being and rights of those who will inherit the Earth, and that being a good prospective parent means being a good ancestor. For a more in depth analysis of the non-identity problem and why it does not need to stand in the way of thinking through our obligations to future generations in regards to climate change, see, for example, Hedberg (2019) - particularly Part II and the Appendix.
